# Miscellaneous Intelligence

**Published:** 1810-10

**Authors:** 


### MISCELLANEOUS INTELLIGENCE.

The following Lectures, given in the Theatre of Anatomy, Great Windmill-street, commence this month, and terminate in May: the hours of delivering them are so arranged as not to interfere with each other, or with the time of attendance at the Hospital.

Chemistry, every morning, from eight until nine o'clock: by William T. Brande, F. R. S; to commence on Monday, the 8th of October.

Materia Medica, twice in the week, from nine until ten o'clock in the morning; by William T. Brande, F. R. S.; to commence on Monday, the 16th of October.

Theory and Practice of Physic, by John Cooke, M. D. F. A. S. and late Physician to the London Hospital; from nine until ten o'clock, four mornings in the week; to commence on Monday, the [illegible]th of October.

Anatomy, Physiology, Pathology, and Surgery, by James Wilson, F. R. S; and B. C. Brodie, F. R. S; from two until four o'clock in the afternoon as usual; to commence on Monday, the 1st of October.

Surgery,

Surgery, by B. C. Brodie, F. R. S; Assistant-Surgeon to St. George's Hospital, from seven until eight o'clock, three evenings in the week; to commence on Monday, the 8th of October.

A room for Practical Anatomy is open every morning, from eight o'clock until two in the afternoon, under the direction of Mr. Wilson, and Mr. Brodie; where a regular demonstration of the dissected parts is given daily, from one until two o'clock.—Twelve Gratuitous Lectures, on the principal operations of Surgery, are given to the pupils of St. George's Hospital, during the season, by Everard Home, Esq. F. R. S. Serjeant Surgeon to His Majesty, and Surgeon to St. George's Hospital.

---

Mr. Chevalier will commence his next Course of Lectures on the Principles and Operations of Surgery, on Monday, the 15th of October, at eight o'clock in the evening, at his house in South Audley-street, Grosvenor-square.

---

Dr. Squire will commence a Course of Lectures on the Principles and Practice of Midwifery, and the Diseases of Women and Children, on Saturday, the 6th of this Month.

---

Medical and Chemical Lectures at St. George's Hospital, and George-street, Hanover-square, will recommence on Saturday, October 6th at No. 9, George-street, Hanover-square, viz. the Medical Lectures, at eight and the Chemical at nine o'clock in the morning, by George Pearson, M. D. F. R. S. sen. Physician to St. George's Hospital, of the College of Physicians, &c.

---

The Practice of the London Infirmary for Diseases of the Eye will be opened to Students on the first of January, 1811.

---

Dr. Farre and Mr. Benjamin Travers will commence in January next at the London Infirmary, in Charterhouse-square, a Course of Lectures, exhibiting the Changes induced by Disease in the several Organs of the Human Body. The Medical department of the Course will be conducted by Dr. Farre,—the Surgical by Mr. Travers. The whole will be illustrated by Preparations and original Cases.

---

The conclusions drawn by Mr. Davey in his late publication on the Muriatic Acid, will serve to extend and enlighten the theory of chemistry to a greater extent than any of the brilliant discoveries formerly made by this illustrious chemist. The following are his conclusions:

1st. That the oxymuriatic acid is (as far as our knowledge extends) a *simple substance*, which may be classed in the same order of natural bodies as oxygen gas; being determined, like oxygen, to the positive surface in voltaic combinations, and like oxygen, combining with inflammable substances, producing heat and light.

2dly That its combinations with inflammable bodies are analogous to oxides and acids in their properties and powers of combination, but they differ from them in being, for the most part, decomposable by water.

3dly. That hydrogen is the basis of the muriatic acid, and oxymuriatic acid its acidifying principle.

4thly. That the compounds of phosphorus, arsenic, tin, &c. with oxymuriatic acid, approach in their nature to acids, and neutralize ammonia and other salifiable bases.

5thly. That the combination of ammonia with phosphorus, acidified by oxymuriatic acid, is a peculiar compound, having properties like those of an earth, and is not decomposable at an intense red heat.

6thly. That oxymuriatic acid has a stronger attraction for most inflammable bodies than oxygen ; and that on the hypothesis of the connection of electrical powers with chemical attractions, it must be highest in the scale of negative power ; and that the oxygen, which is supposed to exist in oxymuriatic acid, has always been expelled by it from water or oxides.

The French chemists questioned the accuracy of the inferences drawn by Mr. Davy from his electro-chemical researches, respecting the nature of the alkalies and the earths ; maintaining that the metallic bodies obtained from these substances, in place of being simple, as asserted by Mr. Davy, were compounds of the alkalies and earths with hydrogen ; or, in other words, that the new bodies were *hydrurets*. Of this opinion were Gay, Lussac, Thenard, and most of the French chemists. Berthollet among the rest warmly contested the correctness of Mr. Davy's inferences, and maintained the accuracy of the French conclusions. At a meeting however of the French National Institute in the end of June, Messrs. Gay, Lussac and Thenard, read a notice containing the results of a great variety of experiments on the new metals ; from all of which they concluded, after a most rigid investigation, that professor Davy was perfectly correct in his inferences ; and with a degree of frankness honourable to themselves, renounced their former opinion that these new metals are hydrurets.

---

Dr. Adams's next course of lectures on the institutes and practice of medicine, will be given at Dr. Anderson's lecture-rooms, No. 47, Frith-street, Soho, commencing on Monday, October 8, at eight o'clock in the morning. On the same morning at nine o'clock, Dr. Anderson will begin his course of scientific and practical chemistry.

---

Mr. Stevenson, of Great Russel-street, Bloomsbury, who as pupil, is intimately acquainted with the practice of the late Mr. Saunders, is preparing a practical work on a frequent Disease of the Eye.

Professor

Professor Leslie, of Edinburgh, has discovered a new mode of producing artificial cold. Without any expenditure of materials, he can, by means of a simple apparatus, in which the action of certain chemical powers is combined, freeze a mass of water, and keep it for an indefinite length of time in a state of ice. In an hour, he has thus formed a cake of six inches in diameter and three quarters of an inch thick; with very little trouble, he can produce a permanent cold of 90 degrees of Fahrenheit, below the temperature of the air, and might easily push it to more than 100 degrees.

---

M. de Saussure, of Paris, lately made a series of experiments on the combustion of several sorts of charcoal. He found that Cornish plumbago, burned in oxygen gas, yields nothing but carbonic acid gas, and oxide of iron, without any mixture of water, or of hydrogen gas. The purest charcoal next to plumbago, is that produced by decomposing the essential oil of rosemary in a red hot tube. In its combustion, it did not form any notable quantity of water; but it gave out some oxicarburetted hydrogen, though in too small a quantity, for the composition of the acid gas to be sensibly modified by it. From this experiment it appeared, that 100 parts of carbonic acid contain 27·11 of carbon, and 72·89 of oxigen. The combustion of anthracite, previously exposed to a red heat, furnished too perceptible a quantity of water and of hydrogen for the results of this process to be calculated with accuracy, and compared with the preceding. The combustion of box charcoal too, dried by long incandescence, furnished an appreciable quantity of water and oxicarburetted hydrogen.

